# Cardiovascular Disease-Related Health Promotion and Prevention Services by Pharmacists in Saudi Arabia: How Well Are They Prepared?

**DOI:** 10.3390/healthcare11111614

**Published:** 2023-05-31

**Authors:** Sirajudeen Shaik Alavudeen, Vigneshwaran Easwaran, Noohu Abdulla Khan, Krishnaraju Venkatesan, Premalatha Paulsamy, Abubakr Taha Mohammed Hussein, Mohammad Tarique Imam, Ziyad Saeed Almalki, Md Sayeed Akhtar

**Affiliations:** 1 Department of Clinical Pharmacy, College of Pharmacy, King Khalid University, Abha 62223, Saudi Arabia; sshaik@kku.edu.sa (S.S.A.); vbagyalakshmi@kku.edu.sa (V.E.); nakhan@kku.edu.sa (N.A.K.); atmohammeed@kku.edu.sa (A.T.M.H.); 2 Department of Pharmacology, College of Pharmacy, King Khalid University, Abha 62223, Saudi Arabia; kvenkatesan@kku.edu.sa; 3 College of Nursing, Mahalah Branch for Girls, King Khalid University, Abha 61421, Saudi Arabia; pponnuthai@kku.edu.sa; 4 Department of Clinical Pharmacy, College of Pharmacy, Prince Sattam Bin Abdulaziz University, Al-Kharj 16273, Saudi Arabia; m.imam@psau.edu.sa (M.T.I.); z.almalki@psau.edu.sa (Z.S.A.)

**Keywords:** pharmacists, cardiovascular disease, attitude, barrier, prevention, Saudi Arabia

## Abstract

Background: Cardiovascular diseases (CVDs) have been identified as the leading reason for morbidity and mortality in Saudi Arabia. Pharmacists play a major role in CVD prevention and health promotion. We aimed to assess the knowledge, attitudes, and involvement of pharmacists in CVD prevention and evaluate the influence of continuing medical education in CVD-prevention services in Saudi Arabia. Method: A cross-sectional study was conducted to evaluate the involvement of pharmacists in CVD-related prevention services along with their knowledge and attitudes. A 34-item questionnaire was developed and distributed among the participants. Results: A total of 324 responses were included in the study. More than 60% of pharmacists had provided counseling regarding the importance of healthy lifestyles and self-monitoring CVD risk factors. About half of the participants (49.1%) had never received any CVD-related continuing medical education. Overall, more than 60% of the participants reported positively towards their role in CVD prevention. Lack of time (66%) and lack of educational materials and tools (41%) were the top perceived barriers for providing CVD-prevention and health-promotion activities, followed by lack of skills in using tools (36%) and lack of privacy/space (33%). Conclusions: The involvement of pharmacists in the prevention of CVD is limited in this study. Further education and capacity building are required to strengthen pharmacists’ involvement in CVD-prevention and health-promotion activities.

## 1. Introduction

Cardiovascular diseases (CVDs) are the leading cause of morbidity and mortality worldwide. The World Health Organization (WHO) has reported that CVDs contributed to 17.9 million deaths in 2019, equivalent to approximately one-third (37%) of premature deaths in people under 70 years [1]. It has been reported that the deaths in the Gulf Cooperation Council (GCC) countries due to CVD is high, estimated to reach up to 65–78% of total adult deaths and around 73% of all deaths in Saudi Arabia [2].

There are a number of known risk factors play a critical role in the precipitation of CVDs. The common modifiable risk factors that are claimed to be responsible for more than 50% of CVDs are elevated blood pressure, type 2 diabetes mellitus (T2DM), smoking, dyslipidemia, and obesity [3,4]. It is apparent that the patients who have these risk factors are at risk of developing CVDs, such as coronary artery diseases or stroke [2]. Various epidemiological research studies conducted in different countries reveal that there is a drop in CVD events when primary prevention approaches are applied earlier among individuals at risk [5,6,7].

The European guidelines on cardiovascular disease prevention in clinical practice and the ACC/AHA guidelines on the primary prevention of cardiovascular disease recommend assessing the CVD risk factors every 4 to 6 years in adult individuals who do not have an established CVD [8,9]. However, time constraints, stress, depression, inadequate social support, and lack of availability of general practitioners are the main barriers to regular CVD risk assessment [10]. Moreover, the healthy and young population is less likely consults their general practitioners for CVD prevention, and physicians give priority to patients who have acute issues over asymptomatic patients with risk factors for CVD [10].

Among these barriers, difficulty in accessing primary-care physicians is an important factor. Hence, the maximum utilization of community-based preventive care has been proposed [11]. In these models, pharmacists are the key healthcare members and play a vital role in the provision of healthcare services. Pharmacists are easily and frequently accessible healthcare members in most communities who have a successful role in controlling CVD risk factors [12]. The growing role of pharmacists in health promotion and their knowledge of medicines management, screening patient adherence, and ensuring the prevention and optimal management of diseases such as CVD has been demonstrated in several studies [13,14,15]. In Saudi Arabia, some of the professional bodies, such as ‘Saudi Society of Clinical Pharmacy’, are conducting continuing medical education programs on the prevention of CVD. However, there is a paucity of data on the knowledge, attitudes, and involvement of pharmacists in CVD-related health-promotion and prevention services, as well as the influence of continuing medical education (CME) on health services in Saudi Arabia. Hence, this study was conducted to assess the knowledge, attitudes, and involvement of pharmacists in CVD prevention and evaluate the influence of CVD training in CVD-prevention services in Saudi Arabia.

## 2. Materials and Methods

### 2.1. Study Design and Sample Size

A cross-sectional study to evaluate the knowledge and the involvement of pharmacists in the prevention of CVD was conducted between June 2021 and August 2021 among pharmacists in Saudi Arabia. The number of registered pharmacists in Saudi Arabia is around 29,090 [16], and the Raosoft software (Raosoft, Inc., 2007, Seattle, WA, United States) was used for calculating the sample size. Keeping a 5.5% margin error, 95% confidence interval, and the response distribution at 50%, the required sample size was calculated as 307. Allowing for an attrition rate of 5%, 324 participants were included in this study.

### 2.2. Study Instrument

It was a questionnaire-based survey. Elements of the questionnaire were chosen based on the researcher’s academic experience and the available literature [12,15]. A 34-item online questionnaire with 3 domains was designed, developed, and validated using the field pretest method and based on the comments, the questionnaire was refined. The pilot study data were analyzed, and internal consistency was calculated (Cronbach’s alpha). The estimated Cronbach’s alpha coefficient was found to be 0.76. The questionnaire consisted of five sections and was composed of both open-ended and closed-ended questions. The first section of the questionnaire included thirteen items to provide information about the demographics and clinical characteristics of the participants (age, gender, nationality, educational qualification, years of experience, type of pharmacy, number of prescriptions per day, availability of functional sphygmomanometers, glucometers, and CME). The second section consisted of seven questions to assess the participants’ involvement in CVD-related health-promotion activities. The third section included seven questions to determine the attitude toward CVD prevention and health promotion. Section four explored the perceived barriers to providing CVD-prevention services and the fifth section consisted of six questions for the assessment of knowledge of the participants on diagnostic cut-offs of hypertension, diabetes, dyslipidemia, and obesity.

### 2.3. Data Collection

The survey was developed using online Google Forms and was circulated to the participants using social media platforms including Facebook, Twitter, WhatsApp, etc.

### 2.4. Data Analysis

Data analysis was performed using the Statistical Package of Social Sciences Software, Version 25.0 (IBM, Armonk, NY, USA). Descriptive statistics were applied to the categorical variables and represented as frequency and percentages. All the categorical variables and the frequency distribution between the groups for various responses to knowledge, attitude, and the associated barriers were estimated using the Chi-square test. A *p*-value of less than 0.05 was taken as the level of significance between responses.

### 2.5. Ethical Considerations

Ethical approval for this study was granted by the Research Ethics Committee at King Khalid University in Aseer region (ECM#2020-1103). All participants were asked for consent before starting the questionnaire.

## 3. Results

In this study, 387 individuals responded to the survey, out of which only 324 responses (84.75%) were complete and found to be suitable to include. Among the 324 participants, the majority of the participants were under the age of 30, and 75% of them were males. Among the participants, 62.7% were Saudi pharmacists, followed by Egyptians (24.7%), and other nationalities (12.6%). The majority of the participants held a bachelor of pharmacy degree (64.5%), followed by a PharmD degree (26.5%), a diploma in pharmacy (5.9%), and a master of pharmacy degree (3.1%). Moreover, 49.4% of the participants had less than 5 years of experience and about half (49.1%) had never received any CVD-related CME. Almost 75% of the participants showed an interest in receiving CVD-related CME. Nearly half of the participants (48.5%) were working in community pharmacies and the rest were working in government and private hospital pharmacies.

Among the participants, 85% were from the southern region. Regarding the number of prescriptions containing medications for CVD, 40.1% were receiving less than 10 prescriptions a day, 30.2% were receiving 11–20 prescriptions a day, 12.3% were receiving 21–30 prescriptions a day, and 17.3% were receiving more than 30 prescriptions a day. Despite the availability of antihypertensive, antidiabetic, and antidyslipidemic medications in all pharmacies, only around one-third of the pharmacies had functional blood pressure and blood glucose monitoring devices. Their general characteristics are shown in Table 1 and Table 2.

Table 3 describes the involvement of the study participants in the CVD-related health-promotion activities. In our study, 59% of the participants were often or always responding to inquiries of the patients related to CVD prevention, and 41% rarely or never responded. Similarly, only 57% of the participants were often or always providing patients with educational materials for preventing CVD. Additionally, 61% of the participants had rarely or never delivered CVD-related prevention activities, namely screening patients for CVD risk factors (BP, blood glucose, body mass index (BMI), and waist circumference). Moreover, only 24% of the participants reported that they advise patients on when to contact the general practitioner regarding hypertension/hyperglycemia/dyslipidemia control and complications. More than 60% of the participants revealed that they often or always counsel patients regarding the significance of a healthy lifestyle, home monitoring of blood pressure, blood sugar, and the timely measurement of blood lipids/cholesterol to prevent CVD (Table 3).

Table 4 describes the attitude of the participants towards CVD-related health-promotion activities. The majority of the participants ‘strongly agreed’ or ‘agreed’ with all seven attitude-related statements. Almost two-thirds of the participants (>70%) strongly agreed or agreed to three attitude-related items such as ‘pharmacists play a major role in the primary prevention of cardiovascular diseases’ (77%), ‘providing counseling on how to prevent CVD to the patients at risk is my responsibility’ (72%), and ‘providing CVD counseling to the patients can improve my professional state and professional satisfaction’ (70%). Similarly, other items included in the attitude domain received positive responses from more than half of the participants, which include ‘I feel confident and prepared to provide CVD-related health-promotion activities to my patients into my daily practice’ (55%), ‘I am confident enough to discuss all the CVD-related drugs with patients’ (55%) and ‘I have good expertise in handling the medical devices like sphygmomanometer, glucometer, etc.’ (53%). Less than half of the participants (49%) agreed that screening for CVD risk factors is their responsibility (Table 4).

The participants’ attitude towards the primary prevention of CVD based on CME is depicted in Table 5. Many of the pharmacists who had CVD-prevention-related CME showed a positive attitude towards the primary prevention of CVD. For instance, attitude items such as ‘Providing counseling on how to prevent CVDs to the patients at risk is my responsibility’ (*p* = 0.010), ‘I feel confident and prepared to provide CVD-related health-promotion activities to my patients into my daily practice’ (*p* = 0.005), ‘I have good expertise in handling the medical devices like sphygmomanometer, glucometer, etc.’ (*p* = 0.003), and ‘I am confident enough to discuss all the CVD-related drugs with my patients’ (*p* = 0.003) showed statistically significant differences between the two groups.

Table 6 compares the knowledge of participants on the diagnostic cut-off points for HTN, T2DM, BMI, waist circumference, and dyslipidemia based on CME. Irrespective of attending or not attending CME, the majority of the participants were found to have lack of knowledge on the diagnostic cut-offs of most of the risk factors.

Table 7 describes the self-reported barriers of the participants for providing CVD-prevention services based on CME activity. A large proportion of the participants (66%) reported that lack of time is one of the main barriers to CVD-prevention practice in their pharmacy. Following this, a lack of educational materials and tools (41%), lack of skills in using tools (36%), lack of space or private counseling area (33%), lack of communication with other healthcare professionals (30%), and deficiency of pharmacy personnel (26%) are some of the other barriers. A lack of therapeutic knowledge or skills in providing counseling, difficulty in identifying targeted patients, and language were also listed as barriers to CVD-prevention services. Among the barriers tested in this study, a lack of skills in using the tools (*p* = 0.027) and lack of therapeutic knowledge or skills in providing counseling (*p* = 0.013) were significantly associated with CVD training.

## 4. Discussion

To the best of our knowledge, this is the first study in Saudi Arabia that has evaluated pharmacists’ knowledge, attitudes toward the primary prevention of CVDs and the degree of their involvement. The participants in our study have perceived their importance in CVD prevention and health promotion. However, there is a significant gap between their perception and the actual practice.

The participants’ CVD-prevention and health-promotion activities are relatively low when compared to similar studies in Australia and Canada [17,18,19], which indicates a lack of involvement of Saudi pharmacists in CVD prevention and health promotion. This may be due to the professional practice standards that specifically include standards on health promotion and education, counseling, screening and risk assessment, and disease state management in those countries. Integrating CVD-prevention and health-promotion activities into pharmacies should be considered a valuable option in Saudi Arabia, as many studies have confirmed the clinical benefits and practicability of CVD-prevention and health-promotion services provided by pharmacists [20,21,22,23].

Considering the immense pressure on the general physicians in the secondary and tertiary healthcare systems in Saudi Arabia due to the increasing prevalence of CVD, the transition of pharmacists’ role from traditional dispensing to primary prevention of CVD and health promotion is warranted [17,24]. This would allow the physicians available for delivering best clinical care. In our study, only 32.1% and 40.1% of the participants indicated the availability of BP and blood glucose monitoring devices, respectively. Only a quarter of the participants stated that they always emphasize the importance of self-monitoring of cardiovascular risk factors, which is far less than the results of a similar study [25]. Better BP and blood glucose control was found in patients who monitor at home regularly compared with patients receiving usual clinic-based care [26], the pharmacist should always emphasize home monitoring of these BP and blood glucose.

It is unfortunate that about half of our participants had not received any CVD-related CME. In a study conducted in Saudi Arabia to assess physicians’ expectations towards pharmacists in CVD prevention and health promotion, pharmacist-provided patient education was acknowledged by the majority of the physicians [27]. To accomplish effective patient education, standardized training and education programs for pharmacists and pharmacy students regarding direct patient care and prevention of CVD should be established and implemented [25,28,29].

More than two-thirds of the participants stated that they never or rarely advised the patients on the importance of regular CVD risk factors’ screening, and around one-third of the participants never counseled on when to contact the general practitioner. These limitations may be associated with the barriers perceived by our participants including lack of time, privacy/space, therapeutic knowledge, and pharmacists’ CVD training, and are consistent with an Australian study [17].

On the other hand, nearly 75% of our participants indicated that they stressed the importance of lifestyle modifications in preventing or controlling CVD risk factors. This is similar to the findings of a previous study conducted among pharmacists in Qatar [18]. This observation is promising, as the risk of developing CVD can be reduced by implementing a healthy lifestyle, such as one that includes regular physical activity, cutting carbohydrates, increasing vegetables, and restricting salt intake [30,31]. Our participants had very positive attitudes toward their role in CVD-related health promotion and prevention and felt confident and prepared to provide CVD-related health promotion despite their lack of CVD-related training. It shows their willingness to upgrade to a patient-centered role from the conventional pharmacist role, as reported earlier [25]. CME plays a major role in the attitudes of pharmacists toward CVD-related health promotion and prevention. Apart from some items, positive attitudes were found among the pharmacists who had attended CME. This is consistent with a study conducted to evaluate the impact of CME among healthcare professionals in Malaysia on diabetes [32].

Several barriers were reported by the participants for providing CVD-related preventive measures. A lack of time, lack of educational materials, and lack of skills in using tools were found to be the top three barriers. These barriers were reported in previous studies that evaluated pharmacists’ involvement in CVD prevention and health promotion [19,33,34]. To optimize public health in Saudi Arabia, integrating pharmacy practice and pharmacies to enhance CVD-related health promotion is a valuable measure. It is therefore important to overcome all the barriers for the better involvement of pharmacists and build public acceptability for pharmacy services in CVD-related health-promotion activities. The Ministry of Health, Saudi Arabia, should ensure the availability of CVD-related educational materials, current guidelines, and tools in pharmacies to tailor patient care pathways [35]. The lack of privacy/space barrier can be overcome by insisting that pharmacies have a private or semiprivate counseling area. A lack of skills in using tools and lack of therapeutic knowledge or skills in providing counseling are mainly associated with the lack of pharmacist CVD-related training. To provide an advanced level of CVD patient care, Saudi pharmacists should undergo CVD-related professional development sessions. These sessions should provide the pharmacists with the CVD knowledge and skills necessary to expand their role in this area.

## 5. Limitations

There are some limitations in this study. Despite the anonymous collection of data, the study results were based on pharmacists’ self-reports, with the possibility of social desirability bias. Other study limitations include the cross-sectional design of the study and that a large number of the participants were from the southern region of Saudi Arabia. This study can be extrapolated by including an equal number of participants from other regions of Saudi Arabia so that the knowledge and attitude towards CVD prevention and health promotion can be measured and compared.

## 6. Conclusions

The increasing prevalence of CVD and its mortality rates necessitate that Saudi pharmacists expand their role in CVD prevention and health promotion. Despite recognizing their crucial role in this regard, a substantial proportion of pharmacists are lagging in providing basic CVD health-promotion activities. Moreover, the participants perceived several barriers. Adequate steps should be taken by the Saudi Arabian health ministry and other health authorities to educate and motivate pharmacists and overcome barriers, to enhance Saudi pharmacists’ role in CVD prevention.

## Figures and Tables

**Table 1:** Demographic characteristics of the participants (n = 324).

Characteristics	n	%
Characteristics of the pharmacists		
Age		
≤30	197	60.8
31–40	116	35.8
>40	11	3.4
Gender		
Male	244	75.3
Female	80	24.7
Nationality		
Saudi	203	62.7
Egyptian	80	24.7
Others	41	12.6
Educational Qualification		
Diploma in Pharmacy	19	5.9
B. Pharm	209	64.5
M. Pharm	10	3.1
PharmD	86	26.5
Number of years of practice		
≤5	160	49.4
6–10	124	38.3
11–15	36	11.1
16–20	4	1.2
Have you ever completed CVD training?		
Yes	165	50.9
No	159	49.1
Interested to take CVD training		
Yes	240	74.1
No	84	25.9

**Table 2:** Characteristics of the pharmacies (n = 324).

Characteristics	n	%
Type of pharmacy		
Community pharmacy	157	48.5
Government hospital pharmacy	92	28.4
Private hospital pharmacy	75	23.1
Location of the pharmacy		
Southern region	278	85.8
Eastern region	21	6.5
Middle region	13	4.0
Western region	6	1.9
Northern region	6	1.9
Number of prescriptions on a workday		
<30	134	41.4
30–50	96	29.6
51–100	37	11.4
100–200	34	10.5
>200	23	7.1
Number of pharmacists in the pharmacy at any one shift		
1	101	31.2
>1	223	68.8
Average number of prescriptions containing medications for CVD risks		
≤10	130	40.1
11–20	98	30.2
21–30	40	12.3
More than 30	56	17.3
Availability of functional sphygmomanometer		
Yes	104	32.1
No	220	67.9
Availability of functional glucometer		
Yes	130	40.1
No	194	59.9

**Table 3:** Current involvement of the participants in CVD-related health-promotion activities (n = 324).

Health-Promotion Activities Statements	Never n (%)	Rarely n (%)	Often n (%)	Always n (%)
Responding to patient inquiries related to cardiovascular diseases prevention including hypertension, diabetes, overweight, smoking and dyslipidemia	25 (8)	106 (33)	115 (35)	78 (24)
Providing patients with educational materials about cardiovascular diseases prevention	39 (12)	100 (31)	114 (35)	71 (22)
Screening patients for the presence of cardiovascular diseases’ risk factors in the pharmacy (BP, Blood glucose and BMI)	88 (27)	111 (34)	88 (27)	37 (11)
Stressing patients on the importance of self-monitoring of blood pressure, blood sugar and timely measurement of blood lipids/cholesterol	29 (9)	95 (29)	117 (36)	83 (26)
Providing patients with advice or counseling regarding the importance of healthy lifestyles to prevent cardiovascular diseases?	17 (5)	82 (25)	119 (37)	106 (33)
Counseling on when to contact the health care provider regarding hypertension/hyperglycemia/dyslipidemia control and complications	32 (10)	75 (23)	140 (43)	77 (24)
Counseling about the cautions of over-the-counter drugs or herbal products as they relate to hypertension/hyperglycemia/dyslipidemia management	31 (10)	89 (27)	123 (38)	81 (25)

BP—Blood pressure; BMI—Body mass index.

**Table 4:** Attitudes of the participants towards CVD-related health-promotion activities (n = 324).

Attitude Statements	Strongly Disagree n (%)	Disagree n (%)	Neutral n (%)	Agree n (%)	Strongly Agree n (%)
Pharmacists play a major role in the primary prevention of cardiovascular diseases	12 (4)	15 (5)	47 (15)	134 (41)	116 (36)
Providing counseling on how to prevent cardiovascular diseases to the patients at risk is my responsibility	12 (4)	26 (8)	51 (16)	143 (44)	92 (28)
Screening for the presence of cardiovascular diseases’ risk factors is the responsibility of the pharmacist	16 (5)	51 (16)	107 (33)	93 (29)	57 (18)
I feel confident and prepared to provide cardiovascular disease-related health-promotion activities to my patients into my daily practice	10 (3)	34 (10)	103 (32)	103 (32)	74 (23)
Providing cardiovascular diseases counseling to my patients can improve my professional state and increase my professional satisfaction	13 (4)	23 (7)	61 (19)	137 (42)	90 (28)
I have good expertise in handling the medical devices like sphygmomanometer, glucometer, etc.	12 (4)	49 (15)	92 (28)	103 (32)	68 (21)
I am confident enough to discuss all the CVD-related drugs with my patients	22 (7)	35 (11)	89 (27)	105 (32)	73 (23)

CVD—Cardiovascular disease.

**Table 5:** Distribution of attitude toward CVD-related health promotion based on CME activity (n = 324).

Attitude Statements	Response										*p* Value
	Strongly Disagree		Disagree		Neutral		Agree		Strongly Agree		
	Had CME n (%)	Did Not Have CME n (%)	Had CME n (%)	Did Not Have CME n (%)	Had CME n (%)	Did Not Have CME n (%)	Had CME n (%)	Did Not Have CV Training n (%)	Had CV Training n (%)	Did Not Have CV Training n (%)	
Pharmacists play a major role in the primary prevention of CVDs	5 (3)	7 (4)	5 (3)	10 (6)	22 (13)	25 (16)	66 (40)	68 (43)	67 (41)	49 (31)	0.297
Providing counseling on how to prevent CVDs to the patients at risk is my responsibility	4 (2)	8 (5)	14 (8)	12 (8)	18 (11)	33 (21)	70 (42)	73 (46)	59 (36)	33 (21)	0.010
Screening for the presence of CVDs’ risk factors is the responsibility of the pharmacist	10 (6)	6 (4)	14 (15)	27 (17)	54 (33)	53 (33)	48 (29)	45 (28)	29 (18)	28 (18)	0.880
I feel confident and prepared to provide CVD-related health-promotion activities to my patients into my daily practice	3 (2)	7 (4)	16 (10)	18 (11)	40 (24)	63 (40)	65 (39)	38 (24)	41 (25)	33 (21)	0.005
Providing CVDs counseling to my patients can improve my professional state and increase my professional satisfaction	4 (2)	9 (6)	12 (7)	11 (7)	28 (17)	33 (21)	62 (38)	75 (47)	59 (36)	31 (19)	0.016
I have good expertise in handling the medical devices like sphygmomanometer, glucometer, etc.	4 (2)	8 (5)	20 (12)	29 (18)	36 (22)	56 (35)	62 (38)	41 (26)	43 (26)	25 (16)	0.003
I am confident enough to discuss all the CVD-related drugs with my patients	7 (4)	15 (9)	13 (8)	22 (14)	37 (22)	52 (33)	64 (39)	41 (26)	44 (27)	29 (18)	0.003

CVD—Cardiovascular disease; Chi-square test was applied to measure the differences in responses between the groups; *p* value ≤ 0.05 was considered significant.

**Table 6:** Knowledge distribution regarding the diagnostic cut-off for common CVD risk factors based on CME activity (n = 324).

Knowledge Items	Responses	All Participants n (%)	Had CME n (%)	Did Not Have CME n (%)	*p* Value
Diagnostic cut-off for hypertension (correct answer: ≥130/80 mmHg)	Correct response	59 (18.2)	32 (19)	27 (17)	0.574
	Wrong response	265 (81.8)	133 (81)	132 (83)	
Diagnostic cut-off for diabetes mellitus (correct answer: ≥126 mg/dL)	Correct response	108 (33.3)	61 (37)	47 (30)	0.157
	Wrong response	216 (66.7)	104 (63)	112 (70)	
Diagnostic cut-off for obesity (correct answer: ≥30 kg/m^2^)	Correct response	102 (31.5)	65 (39)	37 (23)	0.002
	Wrong response	222 (68.5)	100 (61)	122 (77)	
Diagnostic cut-off for abdominal obesity (male) (correct answer: >102 cm)	Correct response	95 (29.3)	43 (26)	51 (32)	0.233
	Wrong response	229 (70.7)	122 (74)	108 (68)	
Diagnostic cut-off for abdominal obesity (female) (correct answer: >88 cm)	Correct response	144 (44.4)	73 (44)	71 (45)	0.941
	Wrong response	180 (55.6)	92 (26)	88 (55)	
Diagnostic cut-off for total cholesterol (correct answer: ≥240 mg/dL)	Correct response	38 (11.7)	17 (10)	21 (13)	0.417
	Wrong response	286 (88.3)	148 (90)	138 (87)	

Chi-square test was applied to measure the differences in responses between the groups; *p* value ≤ 0.05 was considered significant.

**Table 7:** Distribution of self-reported barriers for providing CVD-prevention services based on CME activity.

Perceived Barriers	All Participants n (%)	Had CME n (%)	Did Not Have CME n (%)	*p* Value
Lack of time	215 (66)	109 (66.1)	106 (67)	0.908
Lack of privacy/space	106 (33)	52 (31.52)	54 (34)	0.639
Lack of personnel	84 (26)	43 (26.1)	41 (26)	0.955
Lack of skills in using the tools	117 (36)	67 (40.6)	50 (31.4)	0.027
Lack of monetary benefits	69 (21)	39 (23.6)	30 (19)	0.295
Lack of official recognition for health-promotion activities	68 (21)	35 (21.2)	33 (21)	0.919
Lack of communication with other health care providers	98 (30)	54 (32.7)	44 (28)	0.322
Lack of tools (educational materials, self-monitoring medical devices, and/or no standard guideline available)	134 (41)	64 (38.8)	70 (44)	0.339
Lack of therapeutic knowledge or skills in providing counseling	84 (26)	53 (32.1)	31 (19.5)	0.013
Difficulty in identifying targeted patients	65 (20)	30 (18.2)	35 (22)	0.389
Language barrier	55 (17)	29 (17.6)	26 (16)	0.769
Others	15 (5)	8 (4.9)	7 (4)	0.849

Chi-square test was applied to measure the differences in responses between the groups; *p* value ≤ 0.05 was considered significant.

## Data Availability

The data presented in this study are available on request from the corresponding author. The data are not publicly available due to participants’ confidentiality.
